# Predictors of violence against health professionals during the COVID-19 pandemic in Brazil: A cross-sectional study

**DOI:** 10.1371/journal.pone.0253398

**Published:** 2021-06-17

**Authors:** Mariá Romanio Bitencourt, Ana Carolina Jacinto Alarcão, Lincoln Luís Silva, Amanda de Carvalho Dutra, Nayara Malheiros Caruzzo, Igor Roszkowski, Marcos Rogério Bitencourt, Vlaudimir Dias Marques, Sandra Marisa Pelloso, Maria Dalva de Barros Carvalho

**Affiliations:** 1 Department of Health Sciences, State University of Maringá (UEM), Maringa, Parana, Brazil; 2 Department of Physical Education, State University of Maringá (UEM), Maringa, Parana, Brazil; Imam Abdulrahman Bin Faisal University, SAUDI ARABIA

## Abstract

**Background:**

The increase in violence against health professionals in the COVID-19 pandemic makes it necessary to identify the predictors of violence, in order to prevent these events from happening.

**Objective:**

Evaluating the prevalence and analyzing the variables involved in the occurrence of violence against health professionals during the COVID-19 pandemic in Brazil.

**Method:**

This is a cross-sectional study conducted online involving Brazilian health professionals during the COVID-19 pandemic. The data were collected through a structured questionnaire (Google Online Form) sent to health professionals on social networks and analyzed through logistic regression by using sociodemographic variables. The set of grouped variables was assigned to the final model when p <0.05. A network was built using the Mixed Graph Models (MGM) approach. A centrality measurement chart was constructed to determine which nodes have the greatest influence, strength and connectivity between the nodes around them.

**Results:**

The predictors of violence in the adjusted regression model were the following: being a nursing technician / assistant; having been working for less than 20 years; working for over 37 hours a week; having suffered violence before the pandemic; having been contaminated with COVID-19; working in direct contact with patients infected by the virus; and having family members who have suffered violence. The network created with professionals who suffered violence demonstrated that the aggressions occurred mainly in the workplace, with an indication of psycho-verbal violence. In cases in which the aggressors were close people, aggressions were non-verbal and happened both in public and private places. The assaults practiced by strangers occurred in public places.

**Conclusions:**

Violence against health professionals occurs implicitly and explicitly, with consequences that can affect both their psychosocial well-being and the assistance given to their patients and families.

## Introduction

The COVID-19 pandemic has infected over 150 million people around the world, and over 14 million people only in Brazil. It has been responsible for more than 3 million deaths worldwide and about 406.000 in Brazil [1]. In order to slow down the spread of the virus, several nations have adopted hygiene measures and social distancing, which are the most effective ways to control the disease [2]. Yet, it all has a considerable impact on the mental health of the population [3].

Front-line health professionals are also vulnerable to the emotional impact of this pandemic due to long working hours, inadequate personal protection equipment (PPE) and risk of contamination [4–6].

Stressful work situations are hampered by the stigma that is a product of prejudice and discrimination. Although communities around the world recognize the crucial role played by health professionals, there is evidence of an increase in situations of violence against them associated with the COVID-19 pandemic. Most cases are due to the risk of contamination that these professionals offer because they are on the front line [7].

Violence at work involving health professionals is a phenomenon that has been researched at an international level and, despite the attention it has been receiving, this topic still represents an important challenge. Studies highlight that health workers are at greater risk of suffering violence when compared to other professional sectors, and the worrying naturalization of this phenomenon reinforces the increase in occurrences and the personal consequences for health care activity [8, 9].

Data provided by the World Health Organization (WHO) report that 8–38% of health professionals worldwide are victims of physical violence at some point in their careers [10]. Nonetheless, the pandemic has aggravated situations of violence against them. There are reports in Brazil and other countries of verbal and physical assaults, acts of discrimination and humiliation, such as name-calling and even expulsion from public transport [11, 12].

In addition to the individual consequences for the professionals, the repercussions of violence can have negative implications for the health sector: absenteeism, compromised quality of care and job abandonment, not to mention increased health costs [13].

We found articles reporting on discrimination and violence against health professionals [7–15]. However, to date, no studies have been found in Brazil using the network analysis to demonstrate the variables involved in the occurrence of violence against health professionals during the COVID-19 pandemic.

The aim of this study was to assess the prevalence and analyze variables involved in the occurrence of violence against health professionals during the COVID-19 pandemic.

## Methods

### Study design

This is an exploratory cross-sectional study conducted online with a questionnaire focused on violence against health professionals during the COVID-19 pandemic. The questionnaire and the consent form were sent through social networks to healthcare workers in Brazil, in October 2020, and it remained available for answers for 15 days.

### Study population

The Brazilian Health System encompasses both the public and private sectors. It is called Unified Health System (Sistema Único de Saúde—SUS), and provides universal health coverage to the population, besides promoting universality, completeness, and free care in all spheres healthcare [16, 17]. In addition, SUS also promotes the financing, provision and use of health services in the private sector [18, 19]. Regarding health professionals, especially when it comes to physicians, they can offer their services to the public and private sectors [20]. Table 1 shows the distribution of professionals in each region in Brazil, according to the National Registration of Health Institutions [21].

**Table 1:** Absolute frequency of some health professionals in each region in Brazil.

Occupation	Region					
	North	Northeast	Southeast	South	Midwest	Brazil
Physicians	20,939	81,476	229,626	73,736	36,156	442,133
Nurses	26,162	89,222	156,490	50,236	27,736	34,9846
Nursing assistants/ technicians	65,144	187,700	421,813	133,490	66,251	874,398
Physiotherapists	5,041	22,096	43,286	15,483	7,639	93,545

Data retrieved from the National Registration of Health Establishments in Brazil, March 2021.

### Sampling procedure

Eligible participants for the study were Brazilian healthcare professionals working in the country, aged over 18, from both public and private institutions (clinics, hospitals, basic health units, emergency care units, ambulatories, healthcare transport etc.). They were invited to complete an online survey entitled “COVID-19 pandemic: the genesis of violence against health professionals”. The access link to the survey was disclosed on social media for 15 days, from October 1^st^, 2020. People willing to participate were recruited using a snowball sampling method, a non-probability sampling technique, commonly used in medical science research [22]. Participation was voluntary and anonymous, and individuals could participate and invite other Brazilian healthcare professionals.

### Questionnaire

First of all, the participant had to read and agree with the informed consent form in order to participate in the research. The questionnaire was revised by six health professionals with experience in scientific research in the area (two physicians, three nurses and one psychologist), and it was previously sent to 10 health professionals in order to make sure that the questions and answers were clear and easy to understand, in addition to checking the time required to complete it. At the end, the responses given by the testers were removed and were not part of the sampling.

Therefore, the survey instrument was the aforementioned questionnaire, which was designed to assess the variables involved in violence against healthcare professionals during the COVID-19 pandemic, and it was organized in three sections:

- Demographic profile: sex, age, race, partner, and children;
- Professional information and workplace characteristics: occupation, education level, monthly income, length of service experience, workload, workplace and personal protective equipment (PPE) availability, COVID-19 patients care, and infection by COVID-19;
- Violence characterization: type of violence, who committed it, where it occurred, and if a related person suffered violence motivated by the participant’s profession.

Violence definition was based on the World Health Organization report on violence and health [23], that is, the use of physical force or not, threatened, or actual, against another person, or against a group or community, that results in injury, death, psychological harm, or deprivation. This study evaluated health professionals that experienced different types of violence in different places and caused by different people. The types of violence were physical and psychological. The latter was subdivided into verbal (cursing, swearing, insulting) and non-verbal (discrimination, harassment, prohibition of entering public places, people avoiding contact / staying in the same place as the health professionals). The places where the aggressions occurred were grouped as public (restaurants, stores, means of transport, streets, entrance halls), private (own house or a friend’s house) and workplaces. The aggressors were subdivided according to the relationship with the health professionals: personal relationship (family members, friends, neighbors), professional relationship (bosses, co-workers, and relatives of patients) and strangers.

### Data analysis

Initially, the collected data were synthesized and organized in an Excel® spreadsheet (Microsoft Office, Microsoft Corporation, USA) and, afterwards, analyzed in a descriptive way through absolute and relative frequencies.

A multiple logistic regression model was used to identify the relationship between independent predictors of the variables “sex, race, age, having children, education, having a partner, profession, length of service experience, monthly income, working hours, if they suffered violence before the pandemic, if they already had COVID-19, if they took care of patients with COVID-19, and if their family members suffered violence for having a health professional as family”; and the outcome “participants who suffered violence during the pandemic”.

At first, the explanatory variables were analyzed and selected independently at a significance level of up to 0.2. As for the final model, it was done in a staggered way, and the explanatory variables that remained associated with the outcome with a significance level lower than 0.05, when considered together, were chosen. This way, adjusted *odds ratios* (OR) were calculated for each variable entered in decreasing order of significance. The software used for logistic regression analysis was the R software (R Core Team 2013). In addition to the regression analysis, the network analysis was used to understand the complex interactions between the sociodemographic variables of health professionals included in the study and the aggression variables (type, aggressor and where the aggression took place). As an analytical method, the network analysis calculates multiple associations between two variables while simultaneously conditioning all other variables available in the network model.

The network models are composed of nodes (circles representing each variable included in the model) and edges (lines that connect the nodes). These edges or lines that connect one node to another represent the statistical association between the variables. These statistical associations between pairs, represented by edges, are measured as an average beta coefficient of logistic regressions. While the color indicates the direction of the statistical association (green denotes a positive association and red refers to a negative association), the thickness of the edges represents the strength of the statistical association between the nodes [24].

In this study, weighted non-targeted network graphs were constructed using dichotomous indicators for each aggression variable. The network was estimated by using the Mixed Graphical Models approach, or MGM, which is appropriate for databases with different types of variables (categorical and continuous) [25].

A centrality measurement chart was constructed to determine which nodes have the greatest influence, strength, and connectivity between the nodes around them. Each variable is evaluated by means of expected influence indicators (Expected Influence—evaluates the magnitude in which each node influences the nodes connected to it); strength (Strength—number of connections / correlations between a node and the others); and connectivity (Betweenness—the ability of a variable to serve as a mediation bridge between other variables). Such measures show the importance of each node in the behavior of the network. Values ​​are presented in a Z score and the scoring scale varies from positive to negative values. The higher the value, the greater the influence of a node on others in the network. The analyses were performed using the R Language for Statistical Computing program (R Foundation, Vienna). Predictability of the nodes was computed by the MGM package (version 1.2.10) [25]. Centrality measurements were performed using the qgraph package (version 1.6.5) [26].

### Ethical considerations

The study was approved by the Ethics Committee of the State University of Maringá, registration number 37712820.4.0000.010. This study presented minimal risks to its subjects since their participation was anonymous. The participants were made aware of the aim of the study, and they all signed an informed consent form.

## Results

**Fig 1:**
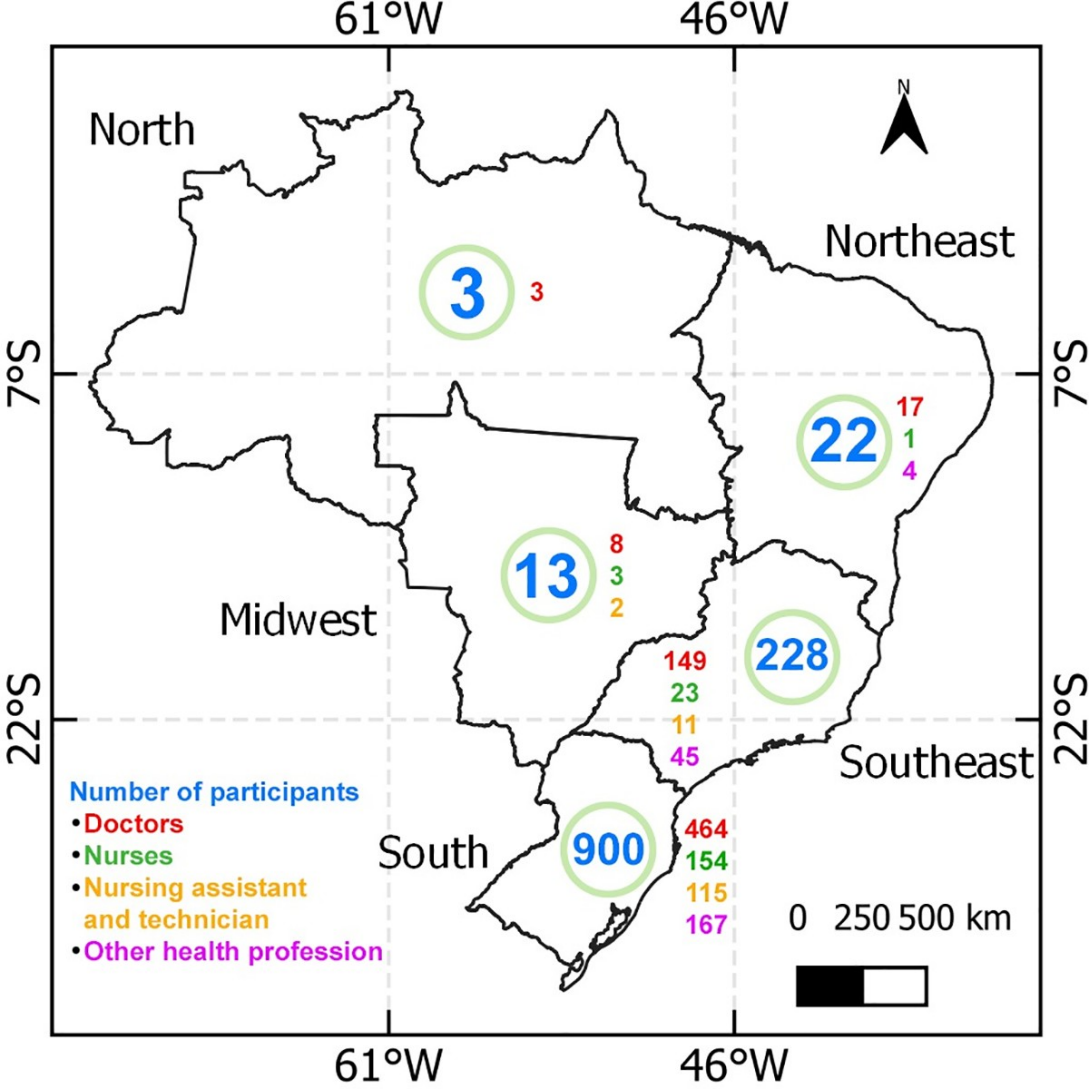
Number of participants, type of profession, and their distribution in each region of Brazil.

The participants of this research were 1,166 Brazilian healthcare professionals from all five regions of the country, most of them from the South. The map of distribution of participants is shown in Fig 1.

The descriptive analysis of the sociodemographic and professional characteristics of the population is shown in Table 2. Notably, most participants were white women, ranging from 18 to 39 years old, parents, not single (they had a partner), physicians, no post-graduation degrees, 11 to 20 years of experience, with an monthly income of up to 20 thousand reals, working over than 44 hours a week, in both public and private institutions (Table 2).

**Table 2:** Sociodemographic and professional characteristics of the research participants.

Characteristics	N	(%)
**Sex**		
Male	288	24.7
Female	878	75.3
**Race**		
White	929	79.6
Black	20	1.7
Others	217	18.7
**Age range**		
18–39	719	61.7
40–59	393	33.7
60–80	54	4.6
**Having children**		
Yes	652	55.9
No	514	44.1
**Education level**		
No academic degree	137	11.7
Undergraduate degree	158	13.6
Residency	694	59.5
Post-Graduation degree	177	15.2
**Partner**		
With a partner	757	64.9
No partner	409	35.1
**Profession**		
Physician	641	54.9
Nurse	180	15.5
Nursing assistant / technician	130	11.2
Others	215	18.4
**Length of service / experience**		
≤ 5 years	307	26.4
6 to 10 years	313	26.9
11 to 20 years	326	27.9
> 20 years	220	18.8
Monthly income* **(Brazilian Reals)**		
≤ 5 thousand	418	35.9
> 5 to 10 thousand	205	17.6
>10 to 20 thousand	295	25.3
> 20	248	21.2
**Weekly workload**		
≤ 36h	300	25.7
37 to 44h	386	33.1
> 44h	480	41.2
**Workplace**		
Public	438	18.3
Private	303	25.4
Public and private	354	44.4
Indefinite**	71	11.9

* US$1 = 5.04 Brazilian reals (according to the dollar exchange rate on June 07^th^ 2021).

** Regarding participants who answered, "other types of places", it was not possible to determine whether they were public or private institutions.

Violence against health professionals during the pandemic was reported by 47.6% of the participants. The risk factors for suffering violence during the pandemic were: not having children or partners, being a nursing assistant or technician, less than 20 years in the activity, a monthly income below 5 thousand Brazilian reals, and working over 36 hours a week (p <0.01) (Table 3).

**Table 3:** Correlation of sociodemographic and professional factors with the risk of violence in the pandemic.

Variable	Violence during the pandemic (N = 1,166)				
	Yes N = 574	No N = 592	OR*	CI 95%	p
	N (%)	N (%)			
**Sex**					
Male	145 (25.3)	143 (24.2)	1.06	0.81–1.38	0.66
Female	429 (74.7)	449 (75.8)	-	-	-
**Race**					
White	450 (78.4)	479 (80.9)	-	-	-
Black	7 (1.2)	13 (2.2)	1.59	0.46–2.73	0.79
Others	117 (20.4)	100 (16.9)	1.18	0.84–1.52	0.38
**Age range**					
18–39	358 (62.4)	361 (61.0)	-	-	-
40–59	182 (31.7)	211 (35.6)	0.87	0.68–1.11	0.27
60–80	34 (5.9)	20 (3.4)	1.71	0.97–3.03	0.06
**Having children**					
Yes	291 (50.7)	361 (61.0)	-	-	-
No	283 (49.3)	231 (39.0)	1.52	1.20–1.91	<0.01
**Education level**					
No academic degree	71 (12.4)	66 (11.1)	-	-	-
Undergraduate degree	83 (14.5)	75 (12.7)	1.03	0.65–1.62	0.90
Residency	317 (55.2)	377 (63.7)	0.78	0.54–1.12	0.18
Post-Graduation degree	103 (17.9)	74 (12.5)	1.29	0.82–2.03	0.26
**Partner**					
With a partner	336 (58.5)	421 (71.1)	-	-	-
No partner	238 (41.5)	171 (28.9)	1.74	1.36–2.22	<0.01
**Profession**					
Physician	304 (53.0)	337 (56.9)	0.85	0.67–1.07	0.17
Nurse	92 (16.0)	88 (14.9)	1.09	0.79–1.50	0.58
Nursing assistant / technician	92 (16.0)	38 (6.4)	2.78	1.87–4.14	<0.01
Others	86 (15.0)	129 (21.8)	0.63	0.47–0.85	<0.01
**Length of service / experience**					
≤ 5 years	196 (34.1)	111 (18.8)	3.70	2.57–5.34	<0.01
6 to 10 years	158 (27.5)	155 (26.2)	2.13	1.49–3.06	<0.01
11 to 20 years	149 (26.0)	177 (29.9)	1.77	1.24–2.52	<0.01
> 20 years	71 (12.4)	149 (25.2)	-	-	-
Monthly income* **(Brazilian Reals)**					
≤ 5 thousand	241 (42.0)	177 (29.9)	1.82	1.33–2.50	<0.01
> 5 to 10 thousand	101 (17.6)	104 (17.6)	1.30	0.89–1.89	0.16
>10 to 20 thousand	126 (22.0)	169 (28.5)	0.99	0.71–1.40	0.99
> 20 thousand	106 (18.5)	142 (24.0)	-	-	-
**Weekly workload**					
≤ 36h	116 (20.2)	184 (31.1)	-	-	-
37 to 44h	190 (33.1)	196 (33.1)	1.53	1.13–2.08	<0.01
> 44h	268 (46.7)	212 (35.8)	2.00	1.49–2.69	<0.01

* US$1 = 5.04 Brazilian reals (according to the dollar exchange rate on June 07^th^ 2021).

Other predictors found were having been infected by the virus, working directly to assist patients infected with COVID-19, and both the health professional and their family members having suffered violence during the pandemic (p <0.01) (Table 4).

**Table 4:** Professional variables and risk of suffering violence in the pandemic.

Variable	Violence during the pandemic (N = 1,166)				
	Yes N = 574	No N = 592	OR*	CI 95%	p
	N (%)	N (%)			
**Violence before the pandemic**					
Yes	182 (31.7)	38 (6.4)	6.77	4.66–9.82	<0.01
No	392 (68.3)	554 (93.6)	-	-	-
**Have you had COVID-19?**					
Yes	93 (16.2)	51 (8.6)	2.05	1.42–2.94	<0.01
No	481 (83.8)	541 (91.4)	-	-	-
**Direct contact with COVID-19 patients**					
Yes	381 (66.4)	274 (46.3)	2.29	1.80–2.90	<0.01
No	193 (33.6)	318 (53.7)	-	-	-
**Family members suffered violence**					
Yes	202 (35.2)	28 (4.7)	10.94	7.21–16.58	<0.01
No	372 (64.8)	564 (95.3)	-	-	-

The statistically significant predictors for the occurrence of violence after an adjusted model were having no partner, being a nursing assistant / technician, having less than 20 years of professional experience, weekly workload equal to or over 37 hours, having suffered violence before the pandemic, being infected with COVID-19, working in direct contact with patients infected with COVID-19, and having family members who have suffered violence (p <0.05) (Table 5).

**Table 5:** Adjusted model of variables to suffer violence during the pandemic.

Variables	Violence during the pandemic		
	aOR*	CI 95%	p
**Partner**			
No partner	1.47	1.05–2.05	0.02
**Profession**			
Nursing assistant / technician	2.32	1.38–3.92	<0.01
**Length of service / experience**			
≤ 10 years	3.22	1.85–5.60	<0.01
11 to 15 years	2.11	1.27–3.49	<0.01
16 to 20 years	1.90	1.20–3.03	<0.01
**Weekly Workload**			
37 to 44h	1.91	1.31–2.79	<0.01
> 44h	1.77	1.18–2.65	<0.01
**Violence before the pandemic**			
Yes	7.14	4.72–10.80	<0.01
**Have you had COVID-19?**			
Yes	1.62	1.08–2.42	0.01
**Direct contact with COVID-19 patients?**			
Yes	1.82	1.35–2.45	<0.01
**Family members suffered violence**			
Yes	11.95	7.55–18.91	<0.01

*aOR: adjusted Odds Ratio.

**Fig 2:**
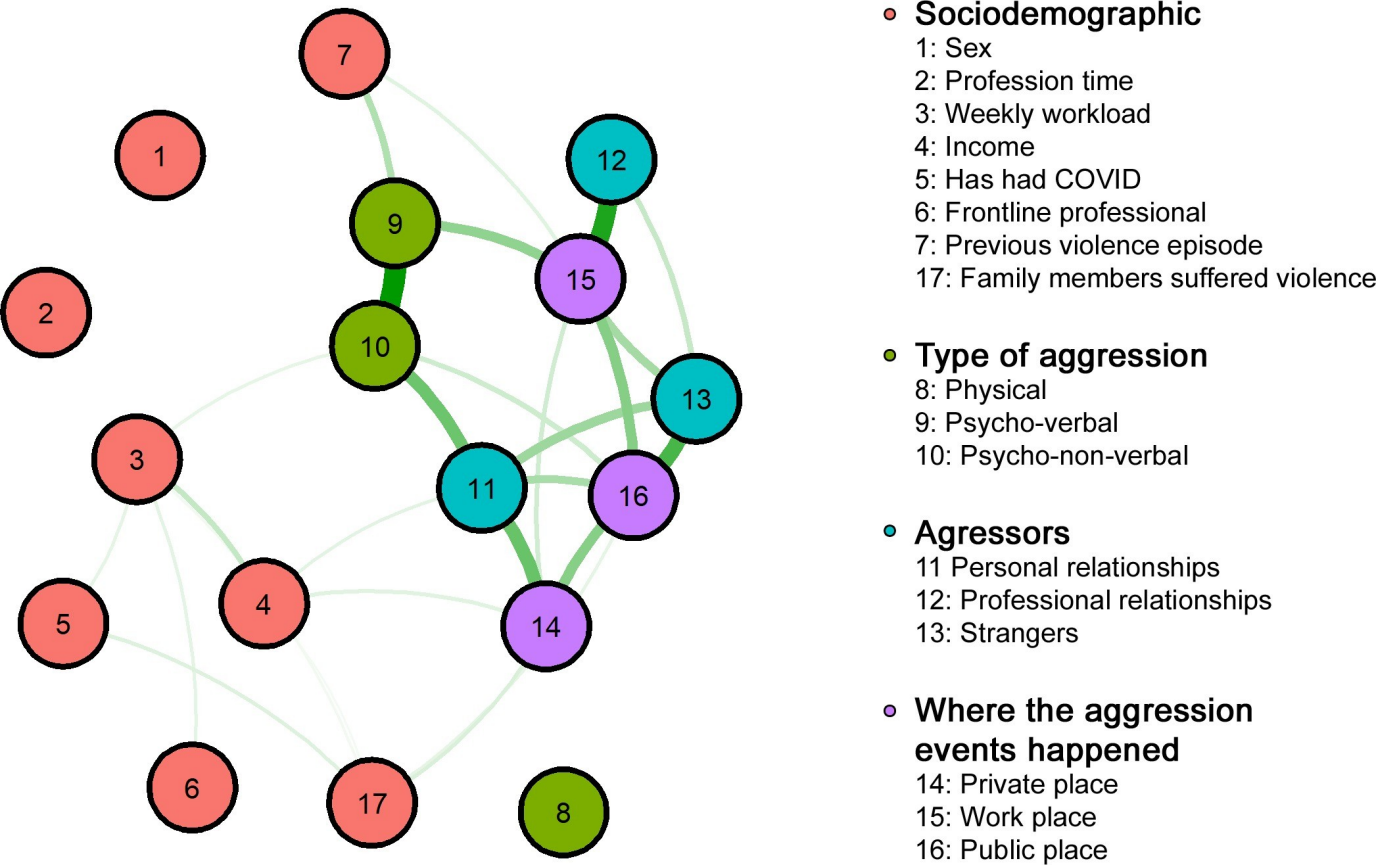
Association network among sociodemographic variables, types of aggression, aggressors, and the place where aggressions against health professionals occurred.

Participants who suffered violence (556) were included in the network analysis with 16 variables: 7 sociodemographic and 9 related to the violence suffered. We identified that physical violence (8) was not associated with other types of violence. We noticed an association between psycho-verbal (9) and psycho-non-verbal (10). The violence practiced by people with professional relationships (12) occurred in the workplace (15), with emphasis on psycho-verbal violence (9). Aggressors identified as people with some personal connections (11) were responsible for non-verbal psychological (9) violence in public (16) and private (14) places, whereas the violence practiced by strangers (13) would occur in public places (16) (Fig 2).

Healthcare professionals who suffered violence prior to the pandemic (7) reported having suffered psycho-verbal violence (9) during the pandemic, in their workplace (15), although it was demonstrated by weak associations. Their monthly income (4) had little association with aggressors with personal relationships (11) and private locations (14). In addition, there was an association between the COVID-19 frontline professionals (6) with working hours (3) and monthly income (4). Infection of the health professionals by the virus (5) showed a weak correlation with weekly workload (3). The other socio-demographic variables did not show significant associations.

**Fig 3:**
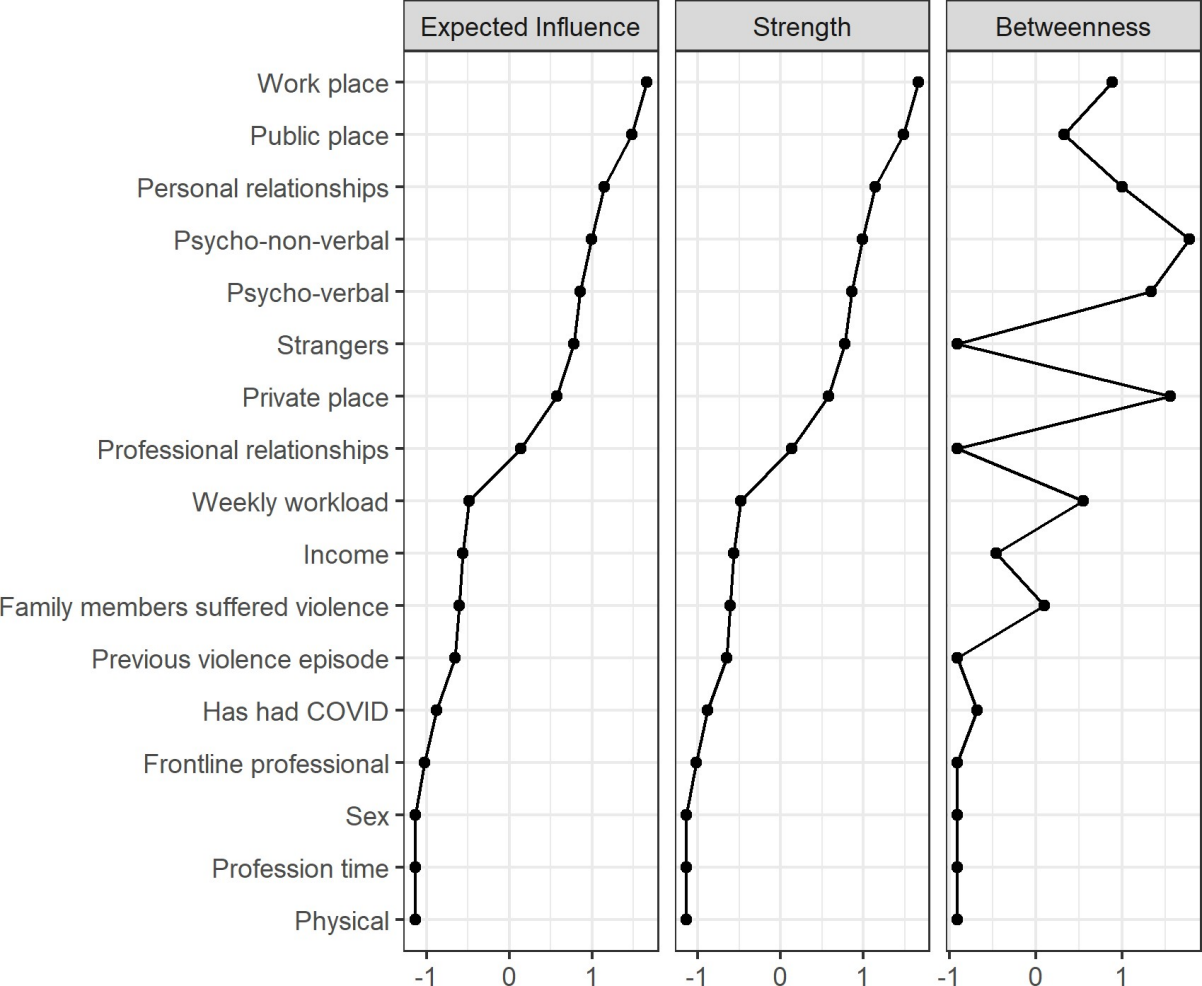
Centrality measures of sociodemographic variables related to the aggression suffered by healthcare professionals.

Regarding the network’s centrality measures, it was noted that the workplace had greater influence and strength compared to the other nodes in the network. Among the types of violence, psycho-non-verbal had greater connectivity, presenting itself as a connecting bridge between psycho-verbal violence and aggressors with personal relationships, which, in turn, demonstrated greater strength between the nodes of the network. That indicates that these variables play an important role in the behavior of the entire network (Fig 3).

## Discussion

This is one of the first studies in Brazil to deal with predictors of violence against health professionals during the COVID-19 pandemic. Almost half of the participants (47.6%) reported having suffered violence during the pandemic. Health workers are 16 times more likely to experience violence at work than other professionals, and hospitals are some of the main places where it occurs [27, 28]. Other types of places include emergency rooms, polyclinics, waiting rooms and geriatric units [29].

The network developed in this study showed that the aggressions occurred mainly at the professional’s workplace, with an indication for psycho-verbal violence practiced by people connected to the professional environment. Other studies have also shown a prevalence of verbal violence by patients and companions of 63.9%, while physical violence corresponded to 7.2% [30]. A study carried out in Taiwan found that 30% of health professionals suffered verbal violence [31].

In this study, only eight health professionals (0.7%) reported having suffered physical violence that did not have any correlations within the network, and the psychological type was the most reported by the participants. There is evidence that people who have been victims of psychological violence are seven times more likely to experience physical violence than those who have not [32]. The consequences of psychological violence against health professionals are as or more severe than those of physical violence. They include decreased job satisfaction, increased occupational stress and worse results in patient care [27].

Although violence against health professionals occurs mainly within institutions [33], there are reports of extramural cases of aggression, as well as violence events on the professionals’ way home or to work [7]. In this study, the network analysis showed that violence at public places was mainly practiced by strangers in a non-verbal psychological way. The fear of being infected by the virus associated with the required distance measures makes people avoid contact with these professionals, often identified by uniforms, lab coats and badges. In Mexico, during the pandemic, some physicians and nurses have been banned from using public transport, in addition to being insulted on the street [14].

Besides the violence practiced by strangers, the network demonstrated that people with personal ties to health professionals were also identified as aggressors. The main type of violence identified was non-verbal psychological violence practiced in public or private places. Close relationships involve more affection and intimacy, which can contribute to perpetuation of violence, since it becomes more difficult to identify and report it. Research on the profile of aggressors in different types of violence has identified them as someone who is well known by the victim, sometimes a family member [34–36].

In addition to the network data, logistic regression identified the main predictors for suffering violence after an adjusted model: being a nursing technician / assistant, having less than 20 years of length of service experience, workload over 37 hours a week, having suffered violence before the pandemic, having been infected with COVID-19, and working in direct contact with patients infected by the virus. Having someone within the family who has also suffered violence was another predictor.

Being a nursing technician or assistant increased the risk of suffering violence during the pandemic (p <0.01). A Brazilian study with nursing professionals pointed to a prevalence of violence among nursing technicians, for these professionals are closer to patients and, thus, more vulnerable to aggression [37].

Professional experience of less than 10 years was the violence predictor with the highest risk among the different lengths of service experience covered by this study (aOR 3.22; p <0.01). There is a relationship between life experiences and the development of a better response in facing the difficulties and uncertainties of clinical practice [38]. Professional experience can have influence on posture, how to deal with conflict situations and interpersonal relationships, as proven by other studies, which found that professionals with longer work experience time were less likely to face violence at their workplace, because they might have better skills in managing conflicts with patients [39]. A study carried out with general practitioners and nurses showed that younger people are more likely to suffer violence at work. As a consequence of their age range, they are the ones with less length of service experience [27].

Working more than 37 (thirty-seven) hours a week tends to increase the risk of suffering violence. Studies have found this same correlation between work overload and violence in the occupational environment [40, 41]. That can favor increased professional stress and decreased quality of patient care [33]. Thus, conflicts can occur involving patients and/or their families [40]. Another study points to high workload as a factor that makes professionals less tolerant towards each other [9].

In this study, having suffered violence before the pandemic represented a risk of suffering violence again during it. Professionals who have experienced some type of aggression in their workplace tend to feel more insecure and dissatisfied in this environment, and to develop unfavorable psychological attitudes towards patients [42]. Passivity in terms of not reporting, and the perception that violence is part of their profession, in addition to lack of support from institutions, can facilitate recurrence. Thus, there is a cycle between having gone through the experience of aggression that interferes with the attitudes of these professionals and, consequently, there are chances of new episodes of violence [43].

The incidence of Coronavirus infection is higher among healthcare professionals due to greater exposure to the virus, especially when it comes to those who work directly with infected patients. Therefore, the risk of suffering violence increases even more because, besides being health professionals, they may have been contaminated [44]. A health professional being infected can raise questions from the population regarding the safety measures in the Health Units and the behavior of all health professionals. Many workers have suffered violence because they were seen as individuals who are more exposed to the virus and, consequently, as carriers who can, thus, spread it. A study carried out with nurses showed that the ones who worked in the emergency and intensive care unit were those who suffered violence the most [45]. These data coincide with the findings of our study, in which front-line professionals, especially those infected, were the most likely to be victims of violence.

Violence against relatives of health professionals is noteworthy in this study for its statistical significance. In Japan, there are reports of health professionals’ children who were bullied, and also forbidden to attend school and take taxis, due to fear of contamination [46].

The pandemic gives rise to a feeling of insecurity in all aspects of life and changes interpersonal relationships [47]. That happens because many people are afraid of contamination and, in order to protect themselves, they avoid contact with members of the families of health professionals.

Unfortunately, violence against health professionals is not a recent phenomenon, and there are reports of violence prior to the pandemic. However, what makes the attacks even more worrying now is that these professionals have been experiencing a crisis that deeply affects society, while being affected by scarce resources, lack of PPE and the risk of contamination [48]. A study carried out during the pandemic in China showed that Wuhan’s front-line professionals were naturally at a high risk of having depression, anxiety, insomnia, distress and stress [49]. That can be further aggravated when they are victims of violence at work. Other symptoms reported as a result of violence at work were fear, decreased self-confidence, sleep disorders, irritability, and panic syndrome [50].

In addition to violence, misinformation must be fought back as it creates erroneous assumptions that can contribute to aggression against these professionals [51]. The use of social media for the dissemination of campaigns combating violence against health professionals is of great importance, both to inform the population with quality data on the disease and to disseminate campaigns to prevent these acts.

This study has some limitations, since it is a cross-sectional study that works with non-probabilistic sampling. The number of participants was significant and included health professionals from all Brazilian regions, allowing the use of different data analysis techniques. The findings cannot be generalized, mainly because these regions are culturally and socio-demographic very different from each other.

## Conclusion

During the pandemic, health professionals have been victims of violence from patients, family members of patients, co-workers, strangers and even their own family members and friends.

Violence against health professionals affects their performance and, consequently, the care provided to patients and their families. Furthermore, suffering violence can trigger or exacerbate stress, anxiety, depression and burnout in professionals and their families during the pandemic.

These data should be the object of future studies in order to implement measures to fight violence and, thus, contribute to the well-being of these professionals, to reducing absenteeism due to mental illnesses and to better patient care.

Aggressions against health professionals affect everyone who depends on their work. Therefore, their well-being is crucial to ensure that the population will be well assisted.
